# The challenge to verify ceramide's role of apoptosis induction in human cardiomyocytes - a pilot study

**DOI:** 10.1186/1749-8090-6-38

**Published:** 2011-03-28

**Authors:** Engin Usta, Migdat Mustafi, Ferruh Artunc, Tobias Walker, Vladimir Voth, Hermann Aebert, Gerhard Ziemer

**Affiliations:** 1 Children's University Hospital, Div. Congenital & Pediatric Cardiac Surgery; University Hospital Tübingen, Germany; 2 Dep. of Thoracic-, Cardiac- and Vascular Surgery; Tübingen University Hospital, Germany; 3 Dep. of Internal Medicine IV, Section of Nephrology and Hypertension; Tübingen University Hospital, Germany; 4 Clinic of Vascular and Thoracic Surgery, Donaueschingen, Germany

## Abstract

**Background:**

Cardioplegia and reperfusion of the myocardium may be associated with cardiomyocyte apoptosis and subsequent myocardial injury. In order to establish a pharmacological strategy for the prevention of these events, this study aimed to verify the reliability of our human cardiac model and to evaluate the pro-apoptotic properties of the sphingolipid second messenger ceramide and the anti-apoptotic properties of the acid sphingomyelinase inhibitor amitryptiline during simulated cardioplegia and reperfusion ex vivo.

**Methods:**

Cardiac biopsies were retrieved from the right auricle of patients undergoing elective CABG before induction of cardiopulmonary bypass. Biopsies were exposed to *ex vivo *conditions of varying periods of cp/rep (30/10, 60/20, 120/40 min). Groups: I (untreated control, n = 10), II (treated control cp/rep, n = 10), III (cp/rep + ceramide, n = 10), IV (cp/rep + amitryptiline, n = 10) and V (cp/rep + ceramide + amitryptiline, n = 10). For detection of apoptosis anti-activated-caspase-3 and PARP-1 cleavage immunostaining were employed.

**Results:**

In group I the percentage of apoptotic cardiomyocytes was significantly (p < 0.05) low if compared to group II revealing a time-dependent increase. In group III ceramid increased and in group IV amitryptiline inhibited apoptosis significantly (p < 0.05). In contrast in group V, under the influence of ceramide and amitryptiline the induction of apoptosis was partially suppressed.

**Conclusion:**

Ceramid induces and amitryptiline suppresses apoptosis significantly in our ex vivo setting. This finding warrants further studies aiming to evaluate potential beneficial effects of selective inhibition of apoptosis inducing mediators on the suppression of ischemia/reperfusion injury in clinical settings.

## Introduction

Cardioplegia and reperfusion of the myocardium are essential techniques employed in many cardiac surgical procedures when a temporarily arrested myocardium is required. However, as a consequence of exposure to cardioplegia and reperfusion apoptosis of cardiomyocytes may occur [1]. Apoptosis is the ultimate result of multiple convergent signalling pathways, which are triggered by events such as nutrient and oxygen deprivation, intracellular calcium overload and excessive reactive oxygen species production [1]. In the setting of cardiac surgery these events can finally result in contractile dysfunction of the myocardium [2] and atrial fibrillation [3]. Apoptosis of cardiac non-myocytes also contributes to maladaptive remodelling and the transition to decompensated congestive heart failure [4]. Regarding this potentially impact of apoptosis on clinical outcomes, there is a demand for pharmacological strategies. Pharmacological blockade has been shown to reduce apoptosis during extracorporeal circulation in an animal model [5]. In contrast to that we have successfully established a human cardiac model, which we have presented recently [6-8].

Our present pilot study was performed just as a sequel to our recent work [6-8] to further evaluate our presented human cardiac model during simulated cardioplegia and reperfusion *ex vivo *respectively the end-points feasibility and reliability. We conducted this study to clarify if another pathway of apoptosis induction in cardiomyocytes exists. Our aim was to evaluate during *ex vivo *simulated cardioplegia and reperfusion the effect of the sphingolipid second messenger ceramide and the anti-apoptotic properties of the sphingomyelinase inhibitor amitryptiline respectively the end-point apoptosis induction and reduction in cardiomyocytes which to our knowledge has not been described in such an experimental setting yet. The results should clarify if any clinical potential utilization could be favoured.

## Materials and methods

### Ethics declaration

The investigation conforms with the principles outlined in the Declaration of Helsinki. In addition, approval was granted by the Ethics Committee of the Faculty of Medicine of the Eberhard-Karls-University, Tübingen, Germany (approval reference number 40/2007 V).

### Patient characteristics

The study protocol was approved by the ethics committee of the Faculty of Medicine of the Eberhard-Karls-University Tübingen. 20 patients undergoing elective CABG surgery were included in this study and gave informed consent for study participation. Mean patient age was 65 years (range 45-70). Mean body mass index 28 kg/m^2 ^(range 25-32). Mean left ventricular ejection fraction 63% (range 55-75). Mean number of diseased coronary vessels 3 (range 2-3). Mean number of infarctions 1 (range 1-3) in patients history. The basic medication of all patients consisted of β-blockers (Beloc Zok™ 47.5 mg twice per die, angiotensin converting enzyme inhibitors, statins and diuretics. All patients had a sinus rhythm.

### Material

Human tissue was retrieved from the auricle of the right atrium of patients before cardiopulmonary-bypass (CPB) and was processed immediately. Each biopsy was transmuraly divided in thirteen pieces with [0.5 to 1 cm^2 ^]size, which were placed separately in microperfusion chambers with continuous perfusion. Cardiac specimens were outside the body before being mounted and tested in the chamber system for a maximum of 30 min, but during this period the oxygen supply was maintained continuously by bubble-oxygenating the Krebs-Henseleit buffer in the petri dish (Greiner Bio-One, Frickenhausen Germany).

### Chemicals and buffer solutions

The modified Krebs-Henseleit buffer (KH) consisted of 115 mM NaCl, 4.5 mM KCl, 1.18 mM MgCl_2_, 1.25 mM CaCl2, 1.23 mM NaH_2_PO_4_, 1.19 Na_2_SO_4_, 80 mM Glucose, and 10 mM HEPES, pH adjusted to 7.4 at 37°C with NaOH.

### Cardioplegic solution

Cardioplegic solution was prepared on the basis of Ca-free KH consisting of 115 mM NaCl, 4.5 mM KCl, 1.18 mM MgCl_2_, 0.5 mM EGTA, 1.23 mM NaH_2_PO_4_, 1.19 mM Na_2_SO_4_, 80 mM Glucose, and 10 mM HEPES, pH adjusted to 7.4 at 37°C with NaOH. Furthermore, a solution containing 20 mM Tris hydroxymethyl-aminomethane, 60 mmol K^+ ^and anionic polypeptides to the isoionic point was added in a 1:4 proportion to Ca-free KH buffer. This solution served as cardioplegic solution and was administered at 4°C, in analogy to our clinical regimen. The resulting K^+ ^concentration in this mixture was 16.5 mM.

### Ceramide

Sphingolipids are constituents of cellular membranes and of lipoproteins. The common backbone is the long chain amino base sphingosine (*trans*-4-sphingenine), and the ceramides refer to the *N*-acyl derivatives of sphingosine. For a decade now, ceramides have been widely studied as regulators of major cellular functions, i.e., apoptosis, proliferation, or senescence [9-11]. Apoptosis induction with short chain ceramide (20-50 μM) supports the view that ceramides are able to trigger apoptosis [12]. The concentration of ceramide employed in this study was 50 μM, similar to previous experimental settings [12].

### Amitryptiline

Amitryptiline (systematic taxonomy: 3-(10,11-dihydro-5H-dibenzo[[a, d]]cycloheptene-5-ylidene)-N, N-dimethyl-1-propanamine) is a tricyclic antidepressant. Besides its known clinical use it has been identified as an acid sphingomyelinase inhibitor with lowering ceramide levels and thus carrying out anti-apoptotic properties [13,14].

### Cell viability

The viability of cardiomyocytes in tissue samples was assessed by trypan blue exclusion before each experiment. Only samples consisting of ≥ 99% viable cardiomyocytes were further processed in the experiments of this study.

### Microperfusion chamber

**Figure 1:**
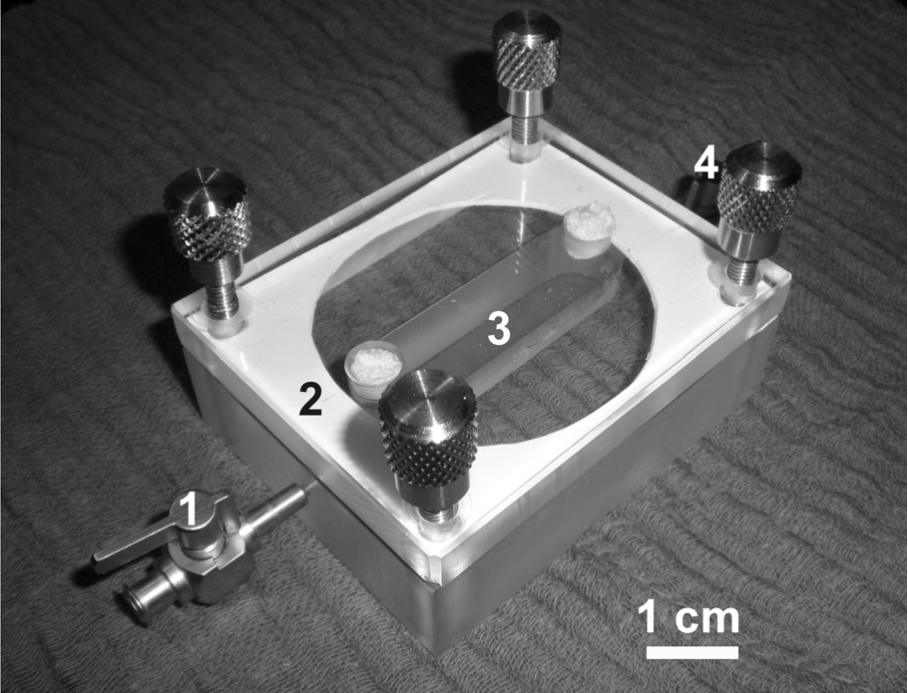
**Microperfusion chamber**. The perfusate enters the chamber, constructed from plexiglas (2), through the pipe (1) and fills the rectangular shaped chamber (3). Once laminar flow is constituted the cardiac tissue is physically fixed before the nylon net (not featured), which spans in a 135° angle. The fluid exits on the opposite side (4). Between the bottom and the upper part of the chamber a rubber layer was placed for sealing and fastened with 4 screws.

Our self developed, previously described [6-8] microperfusion chamber was modified to investigate larger specimens. It consisted of two components (Figure 1). The first component a temperature-controlled plexiglas block contained a rectangular cavity forming the chamber with following dimensions (length × width × height, 5.5 × 1.5 × 1.25 cm). The second component was mounted over the first, and consisted of another plexiglas block forming the ceiling of the chamber. In this chamber nylon net with a pore size of 400 μm was mounted diagonally. To enable perfusion of the chamber, a thin pipe was introduced at one end of the plexiglas component, entered the chamber and exited at the other end. A thin rubber layer between each component sealed the microperfusion chamber. The biopsy was fixed physically at the nylon net by the laminar flow (perfusion velocity of 5 ml/min) of the hydrostatic perfusion system through the chamber.

### Experimental groups

The protocol was designed to simulate clinical routine procedures administering cardioplegic solution with the same K^+ ^concentration (16.5 mM) and temperature (4°C). Five different groups (I - V) were arranged as follows: I (untreated control, n = 10), II (treated control cp/rep, n = 10), III (cp/rep + ceramide, n = 10), IV (cp/rep + amitryptiline, n = 10) and V (cp/rep + ceramide + amitryptiline, n = 10). In group III cardiomyocytes were continuously treated with 50 μM ceramid. In In group IV cardiomyocytes were continuously treated with 100 μM amitryptiline. In contrast to that in group V cardiomyocytes were continuously treated with both drugs ceramid [50 μM] and amitryptiline [100 μM]. In general, each assay was carried out with the specimens of one patient, i.e. specimens of patients were analysed separately.

### Ischemia/reperfusion assay

The cardiac specimens in the microperfusion chambers were initially equilibrated with KH for 5 min (32°C and continuously bubble-oxygenated with carbogen (95% O_2 _and 5% CO_2_) to attain a PO_2 _of 25-30 kPa and pH 7.4. After that the cardioplegic solution (4°C) was administered for 5 min. To induce ischemic injury during the cardioplegia period the perfusion of the microperfusion chamber was stopped and the oxygen supply was discontinued. The cardiac specimens were subjected to various periods of cardioplegia (30, 60 or 120 min) followed by 1/3 of the chosen cardioplegia time as reperfusion (10, 20 or 40 min), as in our surgical routine. For reperfusion 35°C KH was used. Finally, the cardiac specimens were snap-frozen in liquid nitrogen.

### Immunohistochemical apoptosis detection

The slides with the cryosections of the samples (10 μm) were processed prior to the staining according to the manufacturer's recommendation (Epitomics, Inc., Burlingame, CA, USA). The described chemicals were purchased from Biochrom, Berlin Germany. In brief, the cryosections were immersed into the staining dish containing the antigen retrieval solution: 9 ml of stock solution A (0.1 M citric acid solution) and 41 ml of stock solution B (0.1 M sodium citrate solution) were added to 450 ml of destillated H_2_O and adjusted to pH 6.0. After warming for 30 min in a rice cooker and cooling down the slides were washed with TBST (Tris-Buffered Saline and 0.1% Tween 20) for 5 min on a shaker. For the inactivation of endogenous peroxidases the slides were covered with 3% hydrogen peroxide for 10 min and later washed with TBST. After that the slides were immersed into the blocking solution (PBS (Dulbecco's Phosphate Buffered Salts) and 10% bovine serum albumin) for 1 hour.

Later the cryosections were incubated overnight in a humidified chamber (4°C) with antibodies against PARP-1 (Anti-Poly-(ADP-Ribose)-Polymerase)-cleavage (Epitomics, Inc.). PARP is a zinc-dependent DNA binding protein that recognizes DNA strand breaks and is presumed to play a role in DNA repair. PARP is cleaved in vivo by caspase-3 [15]. The antibody only recognizes p25 cleaved-form of PARP-1.

On the other hand cryosections were stained with antibodies against activated Caspase-3 (Epitomics, Inc.), also. Caspases are a family of cytosolic aspartate-specific cysteine proteases involved in the initiation and execution of apoptosis. Caspase-3 (apopain, SCA-1, Yama and CPP32) is a member of the apoptosis execution functional group of caspases, and is either partially or totally responsible for the proteolytic cleavage of many key proteins during apoptosis. Caspase-3 is a cytosolic protein found in cells as an inactive 35 kDa proenzyme. It is activated by proteolytic cleavage into two active subunits only when cells undergo apoptosis (3).

Later for detection to each section secondary HRP-conjugated anti-rabbit antibody (Epitomics, Inc.) diluted in the blocking solution per manufacturer's recommendation was applied and incubated for 1 hour at room temperature.

### Fluorescence microscopy

**Figure 2:**
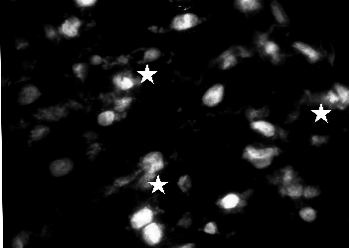
**Representative fluorescent image of cardiomyocytes treated with ceramide during cardioplegia (60 min) and reperfusion (20 min) (group III)**. After DAPI counterstaining the greater nuclei of cardiomyocytes allow their distinction from fibroblasts with smaller nuclei. In anti-activated caspase-3 positive, apoptotic cardiomyocytes the cytoplasm reveales an intensive granular fluorescence (marked with stars). The exemplary images represent a single experiment. During the cryosection procedure artifacts presenting as nuclei conglomerates could not be avoided; these were excluded from analyses.

The number of cells on the cryosections was determined by counting the nuclei of cardiomyocytes after staining with DAPI (4',6-Diamidino-2-phenylindole 2 HCl), a dye known to form fluorescent complexes with natural double-stranded DNA, under a fluorescence microscope (Zeiss, Jena, Germany). In each analysis three different areas of the cryosections were counted using 40-fold magnification. Apoptotic cells were identified by condensation and fragmentation of the nuclei and fluorescent conglomerates in the cytoplasm. They were quantified by counting a total of 200 nuclei from each cryosection and calculating the percentage of apoptotic nuclei. After DAPI counterstaining the greater nuclei of cardiomyocytes allow their distinction from fibroblasts with smaller nuclei. In anti-activated caspase-3 positive, apoptotic cardiomyocytes the cytoplasm reveales an intensive granular fluorescence (Figure 2). In contrast to that PARP-1 cleavage positive, apoptotic cardiomyocytes nuclei feature an intensive granular fluorescence intensity with granular staining of the nucleus.

Fluorescence images (blue) of DAPI loaded cardiac specimens were obtained at an excitation wavelength of 360 nm, with an emission wavelength of 460 nm. DAPI was purchased from Sigma-Aldrich, Germany.

### Statistical Analysis

Analysis of calcium recordings and graphics were obtained using Sigma Plot software (version 9.0, SPSS Inc., Chicago, IL). Data are expressed as the mean±standard error of deviation (SD) and statistical analysis was performed using GraphPad Prism (version 5.0, GraphPad Software, Inc., CA, USA). Comparison of groups was performed using repeated measures one-way ANOVA followed by Tukey's HSD post hoc test. A p value of less than 0.05 was considered to indicate a statistically significant difference.

## Results

### Immunohistochemical apoptosis detection

#### Anti-activated-caspase-3

**Figure 3:**
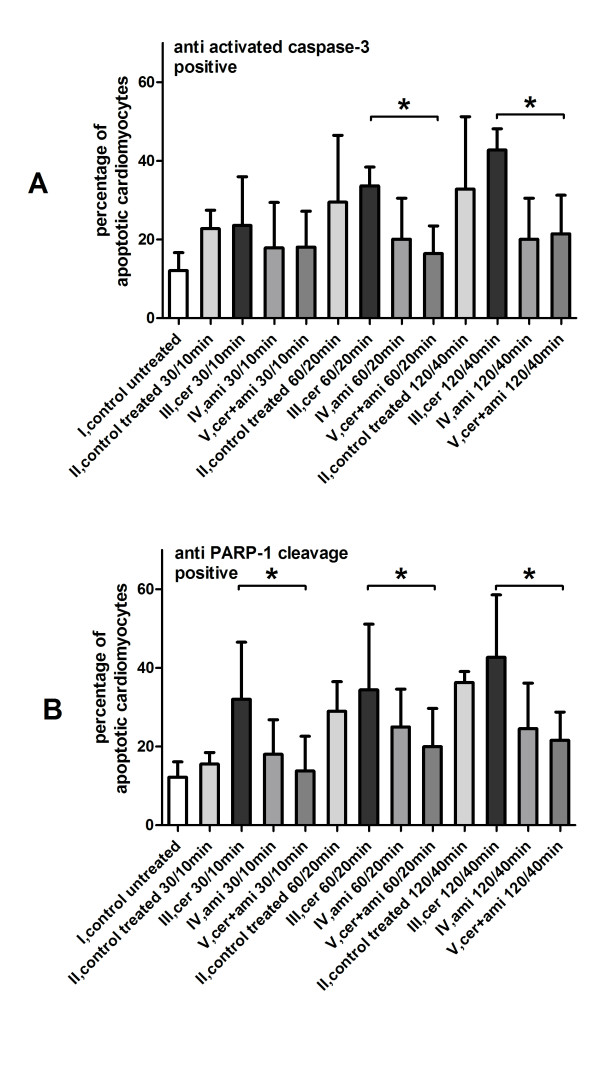
**Demonstrating the effect of ceramide and amitryptline on apoptosis in human cardiomyocytes**. Percentage of anti-activated caspase-3 (3A) and anti-PARP-1 cleavage (3B) positive cardiomyocytes. In the treated control group the time-dependent increase of apoptotic cardiomyoctes is significant (p < 0.05) if compared to the untreated control group. Ceramide had a higher impact on apoptosis if compared to the treated control group. Amitryptiline applied together with ceramide suppressed the proapoptotic effect of ceramide significantly (p < 0.05) (*). Results shown represent mean±SD of combined results from n = 10 independent assays.

Cardiomyocytes in the untreated group I revealed a significant (p < 0.05) low percentage of apoptotic cells (12 ± 5%) in comparison to the treated control group II (Figure 3A). There was a significant (p < 0.05) lower percentage of apoptotic cells in the amitryptiline treatment group IV if compared to group III with ceramide (Figure 3A).

#### PARP-1 cleavage

Cardiomyocytes in the untreated group I featured a significant (p < 0.05) low percentage of apoptotic cells (12 ± 4%) in comparison to the treated control group II (Figure 3B). There was a significant (p < 0.05) lower percentage of apoptotic cells in the amitryptiline treatment group IV if compared to group III with ceramide (Figure 3B).

## Discussion

In the present study our first goal was to apply ceramide to evaluate the proapoptotic potential during cardioplegia and reperfusion [9,16] in an ex vivo setting with human cardiomyocytes which to our current knowledge has not been reported yet. Our second goal was to investigate if the proapoptotic effect of ceramide could be inhibited by amitryptiline [17]. Our third goal was just in accordance to our clinical routine to administer cardioplegia and reperfusion to simulate the extracorporeal circulation in our experimental model and evaluate if the induction or inhibition of apoptosis could be influenced.

In our experimental model human cardiomyocytes were kept in their natural environment as intact cardiac tissue. Otherwise human papillary muscle could be employed but obtaining it before cardioplegic arrest is not an imaginable and feasible option during clinical routine. The simulation of ischemia in isolated cardiomyocyte models can provide important insights into the pathophysiology of myocardial ischemic injury and its underlying molecular mechanisms as was the subject in previous studies in isolated mammalian cardiomyocytes [18], isolated papillary muscle preparations [19] or animal heart models [20]. The distinctive difference of our experimental assay was utilizing human atrial cardiac tissue as a model for apoptosis studies inducing apoptotis just in accordance to our clinical routine with cardioplegia and reperfusion without induction of ischemia with N_2 _perfusion like in previous studies [21,22]. Like presented above in our experimental assay the cardioplegia and reperfusion stimulus proved to be an adequate stimulus for apoptosis induction and is comparable with those in the literature [6-8,23].

Further we wanted to enlighten the major mediators of apoptosis occurring during postischemic reperfusion. Apoptosis is an important mechanism of active cellular death that is distinct from necrosis and has been implicated in the pathogenesis of a variety of degenerative and ischemic human diseases [24]. The family of caspases is key mediator of apoptosis. An extrinsic pathway involving cell surface death receptors [25] and an intrinsic pathway with intracellular and extracellular death signals which are transmitted to the mitochondria through members of the Bcl-2 family [26] exist. Several intracellular stimuli, including oxidative stress, translocate Bax and/or Bak to the mitochondria, leading to dysfunction of this organelle, the release of proapoptotic proteins, and the activation of caspase-9 [27]. Another important stimulus for apoptosis derive from sphingolipids like ceramides which have been described as second messengers for several events like differentiation, senescence, proliferation and cell death in different cell lines [9]. Sphingolipids are found in most subcellular membranes. In the plasma membrane they are predominantly found in the outer leaflet [28]. The metabolism of sphingolipids has been proved to be a dynamic process and their metabolites (such as ceramide, sphingosine, and sphingosine 1-phosphate (S1P)) are now recognized as messengers playing essential roles in cell growth, survival, as well as cell death [9,29]. Sphingomyelin (SM) is a ubiquitous component of animal cell membranes, where it is by far the most abundant sphingolipid. Ceramide can be formed through sphingomyelinases (SMase)-dependent catabolism of SM and by de novo synthesis. SMases are specialized enzymes with phospholipase C activity that can hydrolyze the phosphodiester bond of SM. It is well known that ceramide can modulate many different cellular processes. Ceramide directly regulates protein phosphatase 1 (PP1), inducing dephosphorylation of SR proteins and splicing of caspase-9 and Bcl-x genes [30]. Interaction of ceramide with protein kinase-c can inhibit translocation of the kinase to the plasma membrane and therefore inhibits its catalytic activity. Finally the intrinsic and extrinsic pathways of apoptosis induction converge and lead to the activation of caspases which have been characterized as major executioners of apoptosis [31]. During oxidative stress reactive oxygen species trigger the release of cytochrome c from mitochondria and, subsequently, caspase activation. Active caspases promote cellular demolition by activating other destructive enzymes, such as DNAses, and by directly targeting key structural proteins, such as lamin and actin, and regulatory proteins, thus leading to chromatin margination, DNA fragmentation, nuclear condensation and collapse [31], which we could demonstrate in our immunohistochemical assays.

In our experiments, we found that caspase-3 was already activated at the end of the ischemia, thus suggesting that the mitochondrial pathway of apoptosis is a very early event in myocardial injury. Caspase-3 has been shown to cleave the 112 kDa nuclear protein PARP into an 85 kDa apoptotic fragment [32], and this cleavage by caspase-3 has been shown to be necessary for apoptosis [15]. In this regard, the nuclear presence of proteolytic fragments of PARP has been considered a hallmark of an apoptotic cell. However, the role of PARP-1 in apoptosis remains to be determined because conflicting data have been reported. Some investigators have shown that neurons or hepatocytes from PARP-deficient mice do not exhibit any altered sensitivity to apoptotic stimuli, whereas others have demonstrated that pharmacological or genetic inhibition may increase apoptosis in cells subjected to alkylating agents [33,34]. The family of Bcl-2-related proteins constitutes the most relevant class of apoptotic regulators and, more specifically, the ratio of anti- or pro-apoptotic proteins determines whether the cell will survive or die [35,36]. On the other hand, expression of Bcl-2 protein prevents the induction of apoptosis caused by a variety of oxidative stresses, and it can influence the level of caspase activation [35]

In accordance to this referred data in our presented study we could demonstrate that apoptosis can be suppressed effectively in our experimental setup. Considering our immunohistochemical apoptosis detection there is a significant reduction of apoptosis in cardiomyocytes treated with amitryptiline in contrast to the treatment with ceramide after cardioplegia and succeeding reperfusion. The high apoptosis rate in the treated control group especially after 120 min cardioplegia and 40 min reperfusion should not be extrapolated into the in vivo situation without any caution as atrial and ventricular myocardium possess specific characteristics that may influence the susceptibility to ischaemia/reperfusion injury. One explanation is the reported difference in the distribution of potassium channels [37], which contributes to the characteristic differences between atrial and ventricular action potentials and may determine a different response to cardioplegia/reperfusion.

Our presented data provide evidence that one of the key signaling pathways controlling apoptosis could mediate, at least in part, ischemia-reperfusion induced injury. Furthermore, the results of our study suggest that, although proapoptotic signalling plays an important role in the development of reperfusion-induced damage, acid sphingomyelinase inhibition by amitryptiline aside from dose-dependency may not afford alone a complete protection against postischemic damage. This characteristic has been described in previous studies [14] and could be an explanation for the partial inhibition of apoptosis due to the treatment with amitryptiline like presented in this study.

## Limitations

The present study has few potential limitations. First, clinical ischemia might be quite different from the simulated ischemia we use. Unfortunately, there is currently no accepted standard that constitutes a clinically relevant "simulated ischemic exposure" for cells. Simulating the ischemic environment of the extracellular fluid that bathes the cells is quite complex due to the fact that there are alterations in many factors, simulating all of these events is not currently possible. So, whereas the use of simulated ischemia is not perfect, we believe it recreates a number of the important components of clinical ischemia. Further in this study only a single ceramid and amitryptiline concentration was employed, but analogous to previous studies in a pharmacological relevant concentration [38]. Therefore, detailed dose-response relationships of neither ceramide nor amitryptiline on apoptotic events were not investigated. Nevertheless, with the concentration employed in this study, apoptotic events could be triggered or inhibited considerably. Furthermore the primary purpose of this study was to test its effect on apoptotic events in cardiomyocytes in this new experimental setting rather than to study dose-response relationships. Our next step would be to verify our current findings in an animal model. However our results indicate a definite beneficial effect of amitryptiline on apoptotic events.

## Conclusions

In human cardiomyocytes there is a remarkable induction of apoptosis due to the pro-apoptotic second messenger ceramide.

The treatment of human cardiomyocytes in an *ex vivo *experimental setting with simulated cardioplegia and reperfusion can result in considerable reduction of apoptotic events by adding amitryptiline. These findings warrant further studies in order to evaluate potentially beneficial effects of acid sphingomyelinase inhibition by amitryptiline in the *in vivo *setting of cardioplegia as employed in cardiac surgery.

## Competing interests

The authors declare that they have no competing interests.

## Authors' contributions

EU carried out the routine preoperative examinations, patient evaluation and participated in the study design and coordination. EU performed the statistical analysis. MM, FA and TW participated in the experiments and data evaluation. HA and GZ conceived of the study, and participated in its design and coordination. All authors read and approved the final manuscript.
